# Erythème marginé de Besnier: manifestation rare au cours du rhumatisme articulaire aigu

**DOI:** 10.11604/pamj.2019.32.134.18580

**Published:** 2019-03-20

**Authors:** Bousayna Iraqi, Badr Sououd Benjelloun Dakhamaa

**Affiliations:** 1 Service des Urgences Médicales Pédiatriques, Hôpital d'Enfants, Rabat, Faculté de Médecine et de Pharmacie de Rabat, Université Mohammed V, Rabat, Maroc

**Keywords:** Erythème marginé, rhumatisme articulaire aigu, endocardite infectieuse, Erythema marginatum, acute articular rhumatism, infectious endocarditis

## Abstract

This study reports the case of a 3-year old female child with a 1-year history of rash manifesting as discoid plaques with pink, non-itchy, rounded and oval, polycyclic, confluent macular areas measuring 1-3 cm in diameter, with central clearing evolving in stages, with repeated regression and reappearance. The child was admitted to the Emergency Department with respiratory distress, fever and aggravation of pre-existing skin lesions becoming diffuse. Mitral holosystolic murmur with an intensity of grade 4/6 was recorded by cardiac auscultation. Cardiac ultrasound showed acute massive mitral insufficiency with valvular vegetations. Laboratory tests showed inflammation with a CRP level of 300 mg/l and a sedimentation rate of 60 mm in the first hour and ASLO levels were very high (1600 IU/ml). The diagnosis of infectious endocarditis due to valvular rheumatic heart disease was retained. After stabilization, antibiotic therapy and mitral valve surgery in emergency, the dermatological lesions regressed in a few days but the haemodynamic condition of the child deteriorated rapidly with sudden death. Besnier erythema is a rare cutaneous manifestation of acute articular rhumatism. Physicians should not neglect this rare but useful clinical sign, especially in patients with subclinical valvular involvement in order to avoid potential late cardiac complications. The three differential diagnoses included: ringworm, urticaria, and erythema due to hereditary angioedema.

## Image en médecine

Il s'agissait d'une fillette âgée de 3 ans accusant depuis un an, une éruption cutanée se manifestant par des plaques discoïdes faites de macules rosées, non prurigineuses arrondies et ovalaires, de 1 à 3 cm de diamètre, polycycliques, confluentes et à centre clair évoluant par poussée, avec régression et réapparition. L'enfant a été admise aux urgences dans un tableau de détresse respiratoire et de fièvre avec aggravation des lésions cutanées préexistantes devenant diffuses. L'auscultation cardiaque trouvait un souffle holo-systolique mitral d'une intensité de 4/6. L'échographie cardiaque objectivait une insuffisance mitrale aigue massive avec des végétations valvulaires. Sur le plan biologique, on notait la présence d'un syndrome inflammatoire biologique avec une CRP à 300mg/l et une vitesse de sédimentation à 60 mm à la première heure et les ASLO étaient très élevés (1600 UI/ml). Le diagnostic d'endocardite infectieuse sur valvulopathie rhumatismale était retenu. Après mise en condition, instauration d'une antibiothérapie et d'une cure chirurgicale urgente pour plastie mitrale, il a été noté une régression des lésions dermatologiques en quelques jours mais l'état hémodynamique de l'enfant s'était dégradé rapidement avec un décès brutal. L'érythème de Besnier est une manifestation cutanée rare du rhumatisme articulaire aigu. Les médecins devraient faire attention à ne pas négliger ce signe clinique rare mais utile, en particulier chez les patients présentant une atteinte valvulaire subclinique pour éviter les complications cardiaques tardives potentielles. Les trois diagnostics différentiels étaient l'herpès circiné, l'urticaire et l'érythème de l'angioedème héréditaire.

**Figure 1:**
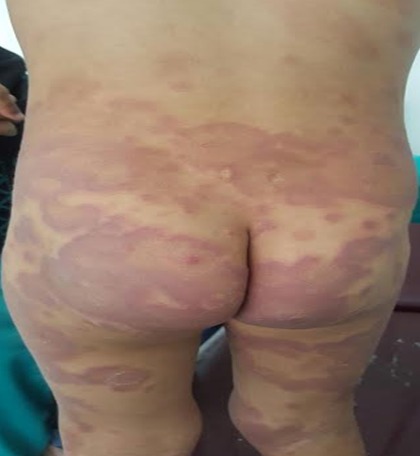
Plaques discoïdes faites de macules rosées, non prurigineuses arrondies et ovalaires, de 1 à 6 cm de diamètre, polycycliques, confluentes et à centre clair siègeant au niveau du siège, du dos et de la face postérieure des cuisses

